# Novel TMS for Stroke and Depression (NoTSAD): Accelerated Repetitive Transcranial Magnetic Stimulation as a Safe and Effective Treatment for Post-stroke Depression

**DOI:** 10.3389/fneur.2020.00788

**Published:** 2020-08-11

**Authors:** Jessica Frey, Umer Najib, Christa Lilly, Amelia Adcock

**Affiliations:** ^1^Department of Neurology, West Virginia University, Morgantown, WV, United States; ^2^Department of Biostatistics, West Virginia University, Morgantown, WV, United States

**Keywords:** post-stroke depression, transcranial magnetic stimulation, stroke recovery, neurostimulation, ischemic stroke, neurorehabilitation, accelerated TMS

## Abstract

**Background:** Post-stroke depression (PSD) affects up to 50% of stroke survivors, reducing quality of life, and increasing adverse outcomes. Conventional therapies to treat PSD may not be effective for some patients. Repetitive transcranial magnetic stimulation (rTMS) is well-established as an effective treatment for Major Depressive Disorder (MDD) and some small trials have shown that rTMS may be effective for chronic PSD; however, no trials have evaluated an accelerated rTMS protocol in a subacute stroke population. We hypothesized that an accelerated rTMS protocol will be a safe and viable option to treat PSD symptoms.

**Methods:** Patients (*N* = 6) with radiographic evidence of ischemic stroke within the last 2 weeks to 6 months with Hamilton Depression Rating Scale (HAMD-17) scores >7 were recruited for an open label study using an accelerated rTMS protocol as follows: High-frequency (20-Hz) rTMS at 110% resting motor threshold (RMT) was applied to the left dorsolateral prefrontal cortex (DLPFC) during five sessions per day over four consecutive days for a total of 20 sessions. Safety assessment and adverse events were documented based on the patients' responses following each day of stimulation. Before and after the 4-days neurostimulation protocol, outcome measures were obtained for the HAMD, modified Rankin Scale (mRS), functional independence measures (FIM), and National Institutes of Health Stroke Scales (NIHSS). These same measures were obtained at 3-months follow up.

**Results:** HAMD significantly decreased (Wilcoxon *p* = 0.03) from M = 15.5 (2.81)−4.17 (0.98) following rTMS, a difference which persisted at the 3-months follow-up (*p* = 0.03). No statistically significant difference in FIM, mRS, or NIHSS were observed. No significant adverse events related to the treatment were observed and patients tolerated the stimulation protocol well overall.

**Conclusions:** This pilot study indicates that an accelerated rTMS protocol is a safe and viable option, and may be an effective alternative or adjunctive therapy for patients suffering from PSD. Future randomized, controlled studies are needed to confirm these preliminary findings.

**Clinical Trial Registration:** https://clinicaltrials.gov/ct2/show/NCT04093843.

## Introduction

The interplay between depression and cerebrovascular disease is complex and clinically important. Post-stroke depression (PSD) is the most common neuropsychological complication of stroke, with a prevalence of ~33% (1) in stroke survivors. PSD adversely influences outcomes by reducing quality of life, increasing caregiver burden, and increasing early mortality as much as ten-fold (2–4). As acute stroke interventions continue to improve, stroke survivorship and associated morbidity will also increase, making the need to explore innovative treatments for PSD even more urgent.

Despite the significant clinical burden of PSD, there are limited treatment options to prevent or reduce its severity. Psychotherapy and pharmacotherapy are well-established as treatments of choice in major depression, however a subset of patients do not respond to either of these first-line therapies (5). Selective Serotonin Reuptake Inhibitor (SSRI) use has been associated with increased risk of hemorrhagic complications as well as increased risk of falls in the elderly, while other studies have shown that SSRIs are actually associated with increased risk for stroke, myocardial infarction, and all-cause mortality (6). A recent meta-analysis for stroke patients concluded that antidepressants did not significantly improve patients' general recovery, achieved varied response rates, and were not tolerated due to adverse effects (7). Compliance, communication problems, and lack of access to psychiatric care are further challenges to treating PSD.

Repetitive transcranial magnetic stimulation (rTMS) may represent an effective treatment option that mitigates the issues associated with the standard PSD interventions. The FDA approved rTMS for patients with Major Depressive Disorder (MDD) in 2008 (8). The typical rTMS protocol that has been used effectively for major depression is 5 days per week for 4–6 weeks. Conventional rTMS paradigms have been studied in the PSD population, and many studies including a meta-analysis have shown that conventional rTMS is likely effective for chronic, refractory PSD (9, 10). However, these conventional paradigms may be inconvenient for patients with limited transportation access and may limit compliancy of patients. Therefore, an accelerated protocol which minimizes the number of days needed to complete the full treatment may be more accessible to patients and may increase compliancy. While there have been some accelerated rTMS paradigms that have been designed to treat conditions such as alcohol withdrawal and treatment-resistant depression (11–14), similar accelerated protocols have not been studied in patients suffering from PSD. Applying accelerated rTMS to the PSD population comes with unique and complex factors. For example, the theoretical risk of seizure using an accelerated protocol may be higher, and this risk may increase even further in patients in the acute to subacute stroke period. Therefore, it is important to study the safety of an accelerated protocol in this population. In addition, the period immediately following cerebrovascular ischemia potentially represents a biologically unique phase amenable to intervention given that both neuroplasticity as well as recurrent stroke risk are highest during this time (15, 16).

There is a clear medical need to further address the impact of rTMS for PSD and to optimize stimulation parameters. We hypothesized that an accelerated 4-days rTMS protocol would be a safe and viable method for treating PSD and would help ameliorate depressive symptoms.

## Methods

This prospective open label study was approved by our Institutional Review Board (IRB # 1804090922) and the Food and Drug Administration granted this study an Investigational Device Exemption (IDE) Number: G180102. The raw data supporting the conclusions of this article will be made available upon request, without undue reservation.

### Participants

All patients admitted to the inpatient stroke service at our tertiary comprehensive stroke center are routinely screened for depression. Patients were screened for depression with the Hamilton Depression Rating Scale (HAMD-17). Study patients were identified either during their acute hospitalization or their follow up clinic visit. Patients who met the inclusion criteria and were otherwise free from the exclusion criteria were eligible to enroll (Table 1). Patients were eligible if the stimulation protocol could be applied between 2 weeks to 6 months following their acute stroke. Between November 2018 and March 2019, 62 of the 98 screened patients fulfilled the inclusion criteria. Although 62 patients were eligible, several patients had logistical issues unique to their own family or social situation and were unable to participate. Six patients were successfully enrolled and completed the stimulation protocol.

**Table 1:** List of inclusion and exclusion criteria.

**Inclusion criteria**
1. Aged 22-85 years old 2. Radiographic evidence of ischemic stroke 3. Stroke within 2 weeks to 6 months 4. HAMD score ≥ 8 **Exclusion criteria**
1. Metallic objects or neurostimulators implanted intracranially 2. Stroke in the area of stimulation (L DLPFC) 3. Known history of epilepsy or seizure disorder 4. A woman who is pregnant or breastfeeding 5. History of psychiatric hospitalization unrelated to current PSD 6. Current suicidal ideation or MINI suicide scale > 8 7. ASRM score > 6 8. Current illicit drug use 9. History of head trauma resulting in loss of memory > 5 min or requiring hospitalization 10. Evidence of hemorrhage in the brain at the time of study 11. Clinically significant EKG abnormalities including QTC prolongation > 450 msec in men or > 480 msec in women 12. Any other mental or physical conditions that are inappropriate for study participation at the PI's discretion

### Stimulation

Neurostimulation was performed using the Neurostar system 2.0 figure of eight coil (Neuronetics, Malvern, PA). Prior to stimulation sessions, patients that were successfully enrolled had additional survey tools administered for baseline assessments in the following categories: modified Rankin Scale (mRS) to assess level of independence, Functional Independence Measures (FIM) to assess quality of independent lifestyle, and HAMD to assess level of depression. Patients were also assessed with the National Institutes of Health (NIH) Stroke Scale to determine physical disabilities resulting from their stroke. All functional scales were performed by trained study personnel and the same rater for each patient was used to minimize variability and inter-rater bias. Vital signs including an electrocardiogram (EKG) were performed before and after each stimulation session. Patients were surveyed about adverse events following each stimulation day.

On the first day, patients underwent a mapping procedure to determine the patient's individualized and optimal Resting Motor Threshold (RMT) over the left motor cortex. The RMT was defined as the minimum stimulation intensity required for visual muscle twitch of the right abductor pollicis brevis (APB) muscle in five out of 10 consecutive single pulse stimulations. After establishing RMT, the coil was moved 5.5 cm anteriorly to the patient's left dorsolateral prefrontal cortex (DLPFC). Patients underwent repeat mapping if necessary. The NeuroStar system has a method for saving each patient's measurements in the system to ensure that the coil is positioned in the same place for each new session. Earplugs were used to prevent any hearing injury. All mapping and treatment sessions were performed by TMS-certified nurses and physicians at our Behavioral Medicine facility where emergency equipment was readily available.

Patients sat in the NeuroStar system chair for all treatment sessions, which has mechanisms to keep the patient properly positioned for mapping and stimulation sessions. The treatment protocol was adapted from other accelerated rTMS protocols in the literature for other indications (11, 12). The protocol included high frequency (20 Hz) rTMS applied over the left DLPFC at 110% RMT for five sessions per day, over four consecutive days for a total of 20 sessions. Forty trains of two second duration were applied with a 12 second intertrain interval for a total of 1,560 pulses per session. Patients were given the opportunity to rest for 10–15 min in between sessions. The treatment sessions lasted for about an hour and a half each day. Variations on the accelerated paradigm we used in this study using different frequencies and different trains may be possible to test in future studies.

At the end of the 4 days of stimulation, patients were once again surveyed with the HAMD, mRS, and FIM. Post-treatment NIH was also performed. The patients were also surveyed at the end of each stimulation day as well as at the end of all 4 days regarding any adverse events they may have experienced. These same measures were once again repeated at the patient's 3-months follow-up.

The primary outcome of this study was safety and viability as defined as the successful recruitment and treatment of participants using the outlined accelerated protocol with no significant adverse effects observed. The secondary outcome was any effect on depressive symptoms as measured by the HAMD. We defined a meaningful response as remission of depression to non-depressed range (HAMD < 8) or at least a 50% reduction in overall score.

### Statistical Analysis

All analyses were conducted in SAS 9.4. Categorical variables are described with frequencies and valid percentages, continuous variables with means and standard deviations. Alpha was set to 0.05 unless otherwise noted. Differences were explored using Wilcoxon signed rank tests on the differences between pre- and post- for continuous variables. Symmetry tests and McNemar's exact tests were run on the ordinal and binary outcome data. Finally, associations were examined between continuous data using Pearson correlations, and with categorical data using Wilcoxon two-sample tests with two-sided t-approximation.

## Results

Demographically, five of the study participants were male and the average age was 66.33 (range 57–71). Stroke etiology included two large artery atherosclerosis (LAA), one small vessel disease (SVD), two cardioembolic (CE), and one embolic source of unknown significance (ESUS). Half of the patients were taking SSRIs at the time of the study (Table 2).

**Table 2:** Baseline characteristics of the six participants.

**Variable**	**Mean or N**	**SD or %**
**Age**		
Years	66.33	4.97
**Gender**		
Male	5	83.33%
HLD^*^		
Yes	5	83.33%
**DM^†^**		
Yes	2	33.33%
**AF**^**‡**^		
Yes	1	16.67%
**Tobacco**		
Yes	3	50.00%
SSRI^§^		
Yes	3	50.00%
**Family history**		
Yes	1	16.67%

* *HLD, hiperlipidemia*.

† *DM, diabetes mellitus*.

‡ *AF, atrial fibrillation*.

§ *SSRI, serotonin selective reuptake inhibitor*.

No significant adverse events related to the treatment were observed. All participants tolerated the stimulation well. One subject described a headache that was milder than his usual chronic headaches and another subject experienced transient facial sensitivity ipsilateral to the coil at the beginning of the first day of stimulation. Neither of these observations were rated as bothersome by the participants and both were self-limited.

HAMD significantly decreased (Wilcoxon *p* = 0.03) from M = 15.5 (2.81) to 4.17 (0.98) following rTMS, a difference which persisted at the 3-months follow-up (*p* = 0.03). There was no statistically significant difference in FIM, mRS, or NIH (Table 3).

**Table 3:** Participant outcome measures (*N* = 6).

**Variable**	***N***	**Pre mean (SD)**	**Post mean (SD)**	**3 month Mean (SD)**	**Diff (pre to post)**	*p*-value^*^	**Diff (pre to 3 month)**	*p*-value^*^
HAMD	6	15.50 (2.81)	4.17 (0.98)	3.50 (2.66)	11.33 (2.94)	0.03	12.00 (3.63)	0.03
FIM	6	115.33 (8.12)	122.17 (6.97)	–	−6.83 (4.17)	0.063	–	–
NIHSS	6	1.83 (2.99)	1.00 (1.67)	–	0.83 (2.04)	1.00	–	–
**Variable**	**Category**	**Pre** ***N***	**(%)**	**Post** ***N***	**(%)**	***p***-value^**^		
mRS						0.80		
	0	1	(16.67%)	2	(33.33%)			
	1	4	(66.67%)	3	(50.00%)			
	2	1	(16.67%)	1	(16.67%)			
NIHSS <4	Yes	4	(66.67%)	5	(83.33%)	0.32		

* *Wilcoxon signed rank test*.

** *Symmetry test, McNemar's exact test*.

**Figure 1:**
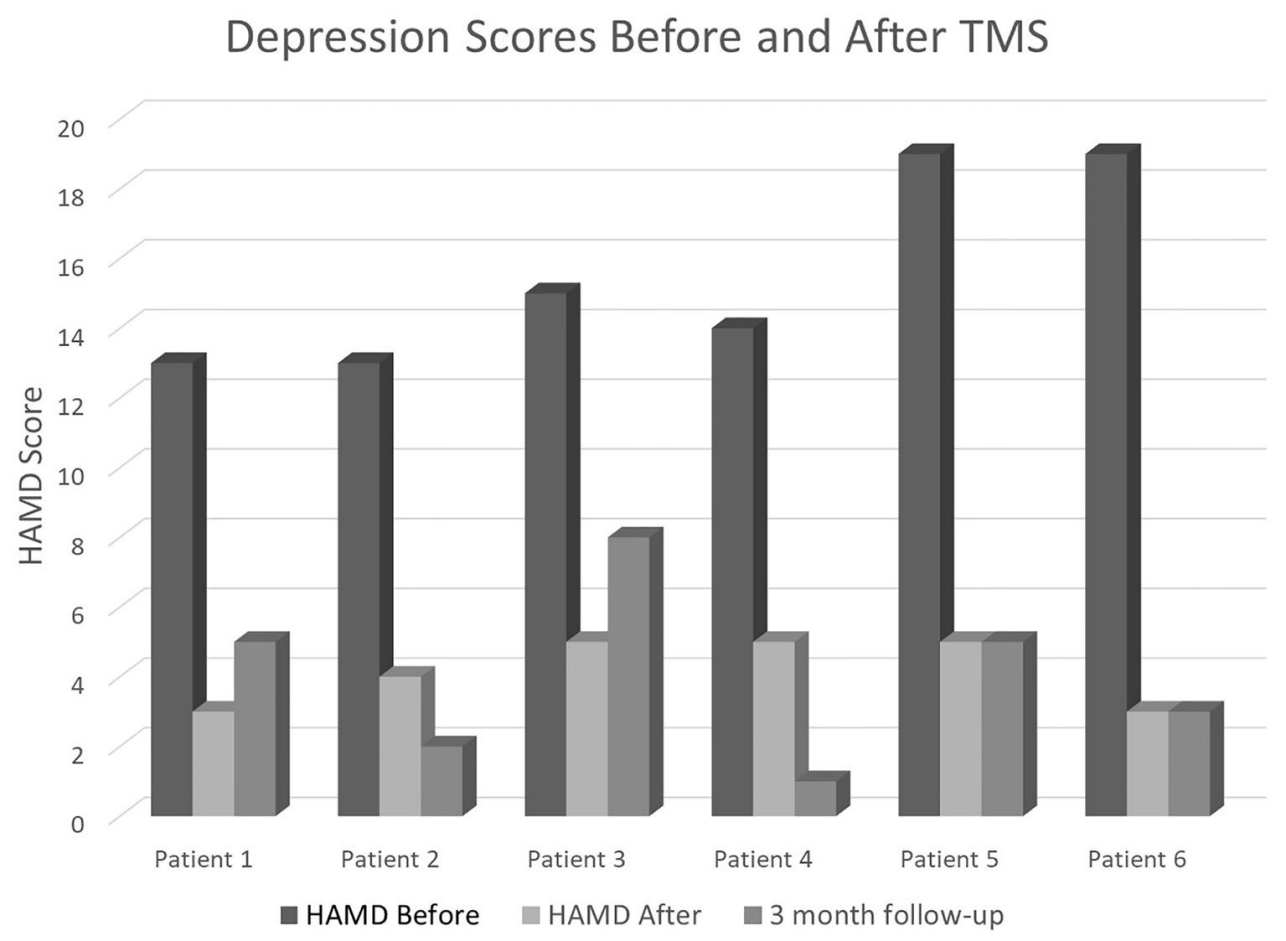
HAMD scores in our six patients before the TMS sessions (baseline), immediately after completing the full 4-days neurostimulation protocol, and at their 3-months follow-up appointment.

In terms of number of patients going from “depressed” (HAMD ≥ 8) to “non-depressed” (HAMD < 8), four participants (66.67%) had moderate depression (HAMD 14–18) and 2 (33.33%) had severe depression (19–22) at baseline. At post-assessment, all scores dropped below the cut-off for non-depressed. At 3-months follow-up, 5 of 6 patients remained non-depressed, and one patient scored eight at the lowest end of mild depression (Figure 1).

## Discussion

Our results demonstrate that the use of an accelerated rTMS protocol in patients with PSD during the subacute period following stroke is a safe and viable option for stroke patients. None of the participating patients reported any significant adverse effects. This high degree of tolerability is similar to the previous published experience with accelerated protocols (11–18). All treated patients experienced a significant improvement in depressive symptoms, with a remission rate of 100% directly following TMS. Remission status persisted in five of the six patients at 3-months follow-up, with one patient scoring borderline mild depressed but still maintaining a 47% reduction in her depression score from baseline.

There have been a few other small studies that have looked at rTMS for chronic PSD (9, 19–21) as well as a recent meta-analysis of 22 randomized controlled trials comparing active rTMS stimulation to sham stimulation (10). These trials indicated that rTMS is an effective tool to treat chronic PSD. Other forms of non-invasive brain stimulation such as electroconvulsive therapy (ECT) and transcranial direct current stimulation have limited data for the treatment of PSD. ECT is largely regarded as the most powerful tool to treat severe depression, however, it is limited by side effects of amnesia (22, 23). Within the PSD population, these findings with ECT are echoed with respective response and remission rates of 60 and 50% (24). In spite of this, rTMS is still the best at controlling frequency and location of stimulation, which offers certain advantages (23). Our data demonstrates that an accelerated version of rTMS may be an effective treatment for PSD as well.

The mechanism underlying rTMS efficacy is still largely unknown. It is hypothesized that low frequency TMS stimulates inhibitory neurons while high frequency TMS stimulates excitatory projection neurons, thus mimicking neuroplasticity through long-term potentiation (23). Thus we chose high-frequency stimulation of the left DLPFC given that this area is associated with depression. However, the translation of cortical excitation to clinical response with rTMS is incompletely characterized (25). Therapeutic benefit is likely achieved through multiple mechanisms enhancing neuroplasticity, increasing available concentrations of critical neurotransmitters, and reinforcing emotionally positive connectivity networks while diminishing connectivity in emotionally negative loops (26–28). Low levels of peripheral and central brain derived neurotropic factor (BDNF) have been observed in depressed individuals as well as those who develop PSD (29–33). Glutamate is emerging as another biomarker for treatment response with increased radiolabeled activity in the DLPFC following stimulation (34). rTMS treatment has also increased dopamine concentrations (35–37), and increased activity within mood networks on functional imaging (38). Exactly how rTMS exerts its influence, however, remains a critical question. Understanding its underlying mechanism will potentially increase our understanding of PSD itself and help identify therapeutic targets.

The novelty of this rTMS paradigm is the accelerated protocol as well as the stimulation in the acute to subacute stroke period. Similar accelerated protocols have been used in other populations (12, 13, 17, 18) (treatment resistant depression and alcohol withdrawal craving) and there have also been studies conducted of rTMS in the acute stroke setting for complications unrelated to depression (39–42); however, a similar paradigm has not yet been employed in a PSD population. A major barrier of current rTMS protocols is the 4–6 weeks timeline before clinical benefit is achieved, so an accelerated protocol is an important potential solution to this problem. The accelerated protocol that was used in this study enabled patients to receive 20 total stimulation sessions, which is the typical minimum number of sessions that patients receive in a conventional rTMS protocol (20 sessions spread out over 4 weeks, receiving one session per day Monday through Friday). Condensing these 20 sessions into four consecutive days allowed patients to participate who otherwise may have faced logistical challenges to obtaining this treatment.

Although this study was underpowered to demonstrate efficacy, the significant remission rate is promising. Larger, randomized studies are needed to confirm these results. There are several limitations in this study. The open label design of this study allows for patients to know they are receiving active stimulation, and the placebo effect could very well have influenced the robust improvement in depression following rTMS. It is important to conduct future trials with a control group and appropriate blinding to truly determine if the rTMS itself is causing a meaningful response in depressive symptoms. Another major limitation is the small sample size. The patients enrolled in the study all had high functional levels according to their FIM, NIHSS, and mRS scores, which may indicate a self-selection bias. It is unclear if patients with a higher functional status were more interested in the study, if these patients were more likely to be aware of their depressive symptoms and want to participate for this reason, or if these patients were more capable of driving themselves to the appointments and thus more willing to participate. In addition, the fact that such a small proportion of eligible patients ultimately enrolled in this study underscores the complexities of treating this patient population and the explicit barriers to enrollment deserve dedicated further study. Regardless, a larger sample size with a group representative of the whole spectrum of post-stroke functionality would allow the results to be applicable to a broader population. In addition, half of our patients were already taking an anti-depressant at the time of enrollment. We chose not to exclude patients on SSRIs since the main goal of this study was to first establish safety and tolerability of using accelerated rTMS in this population, however we did ensure that all patients continued concurrent pharmacologic treatment throughout the duration of the study. Future studies would benefit from excluding patients on SSRIs, and larger studies would also benefit from comparing patients receiving rTMS alone vs. rTMS plus SSRIs to determine if there is a synergistic effect in this population. Similar to major depression, some studies have shown synergism between rTMS and pharmacologic therapy as opposed to either alone (43). However, a meta-analysis of all rTMS in PSD trials published found an rTMS effect size greater among those not on any pharmacologic treatment (0.96) compared to combination therapy (0.51) (22). Future studies may also benefit from the use of neuronavigation to confirm coil position as well as EEG compatible TMS to assess for subclinical seizure activity in a population with a theoretically increased risk of seizure (44). Given the subjective nature of depressive symptom reporting and known placebo effect among depressed patient populations, it is imperative to confirm our findings in larger, randomized studies with a sham stimulation arm as a control group.

## Conclusion

Our results indicate that accelerated rTMS is a safe and viable treatment option for PSD in the subacute stroke population. Depressive symptoms significantly improved in all treated patients. Confirming these results in larger randomized settings has the potential to establish accelerated rTMS as a potent therapy for PSD. Further studies regarding mechanism of action, subgroups particularly responsive to the treatment, and durability of rTMS for PSD are warranted. We are currently conducting a larger randomized controlled study in efforts to answer these questions.

## Data Availability Statement

The raw data supporting the conclusions of this article will be made available by the authors, without undue reservation.

## Ethics Statement

The studies involving human participants were reviewed and approved by West Virginia University Institutional Review Board (IRB). The patients/participants provided their written informed consent to participate in this study.

## Author Contributions

JF was responsible for the conceptual design of this study, writing of the first draft, all major revisions, patient recruitment, data collection, and data analysis. AA was responsible for writing the first draft, major revisions, collaboration of the experimental design, patient recruitment, data collection, and data analysis. CL was responsible for the statistical analysis and revisions to the manuscript. UN was responsible for revisions to the manuscript and contribution to conceptual design. All authors approved the final manuscript.

## Conflict of Interest

The authors declare that the research was conducted in the absence of any commercial or financial relationships that could be construed as a potential conflict of interest.

## References

[1] Villa RF Ferrari F Moretti A. Post-stroke depression: mechanisms and pharmacological treatment. Pharmacol Ther. (2018) 184:131–44. 10.1016/j.pharmthera.2017.11.005 29128343

[2] Bartoli F Lillia N Lax A Crocamo C Mantero V Carrà G. Depression after stroke and risk of mortality: a systematic review and meta-analysis. Stroke Res Treat. (2013) 2013:862978. 10.1155/2013/862978 23533964 PMC3606772

[3] Paolucci S. Epidemiology and treatment of post-stroke depression. Neuropsychiatr Dis Treat. (2008) 4:145–54. 10.2147/NDT.S2017 18728805 PMC2515899

[4] Espárrago Llorca G Castilla-Guerra L Fernández Moreno MC Ruiz Doblado S Jiménez Hernández MD. Post-stroke depression: an update. Neurologia. (2015) 30:23–31. 10.1016/j.nrleng.2012.06.006 22901370

[5] Al-Harbi KS. Treatment-resistant depression: therapeutic trends, challenges, and future directions. Patient Prefer Adherence. (2012) 6:369–88. 10.2147/PPA.S29716 22654508 PMC3363299

[6] Robinson RG Jorge RE. Post-stroke depression: a review. Am J Psychiatry. (2016) 173:221–31. 10.1176/appi.ajp.2015.15030363 26684921

[7] Xu XM Zou DZ Shen LY Liu Y Zhou XY Pu JC. Efficacy and feasibility of antidepressant treatment in patients with post-stroke depression. Medicine (Baltimore). (2016) 95:e5349. 10.1097/MD.0000000000005349 27828858 PMC5106064

[8] O'Reardon JP Solvason HB Janicak PG Sampson S Isenberg KE Nahas Z. Efficacy and safety of transcranial magnetic stimulation in the acute treatment of major depression: a multisite randomized controlled trial. Biol Psychiatry. (2007) 62:1208–16. 10.1016/j.biopsych.2007.01.018 17573044

[9] Jorge RE Robinson RG Tateno A Narushima K Acion L Moser D. Repetitive transcranial magnetic stimulation as treatment of poststroke depression: a preliminary study. Biol Psych. (2004) 55:398–405. 10.1016/j.biopsych.2003.08.017 14960293

[10] Shen X Liu M Cheng Y Jia C Pan X Gou Q. Repetitive transcranial magnetic stimulation for the treatment of post-stroke depression: a systematic review and meta-analysis of randomized controlled clinical trials. J Affect Disord. (2017) 211:65–74. 10.1016/j.jad.2016.12.058 28092847

[11] Baeken C Marinazzo D Wu GR Van Schuerbeek P De Mey J Marchetti I Accelerated HF-rTMS in treatment-resistant unipolar depression: insights from subgenual anterior cingulate functional connectivity. World J Biol Psych. (2014) 12:2014 10.3109/15622975.2013.872295 24447053

[12] Herremans SC Van Schuerbeek P De Raedt R Matthys F Buyl R De Mey J The impact of accelerated prefrontal high-frequency repetitive transcranial magnetic stimulation (rTMS) on cue-reactivity: an fMRI study on craving in recently detoxified alcohol-dependent patients. PLoS ONE. (2015) 10:2015 10.1371/journal.pone.0136182 PMC4546410 26295336

[13] Holtzheimer PE McDonald WM Mufti M Kelley ME Quinn S Corso G. Accelerated repetitive transcranial magnetic stimulation (aTMS) for treatment-resistant depression. Depr Anx. (2010). 27:960–3. 10.1002/da.20731 20734360 PMC3020591

[14] Fitzgerald PB Hoy KE Elliot D Susan McQueen RN Wambeek LE Daskalakis ZJ. Accelerated repetitive transcranial magnetic stimulation in the treatment of depression. Neuropsychopharmacology. (2018) 43:1565–72. 10.1038/s41386-018-0009-9 29467437 PMC5983543

[15] Coleman ER Moudgal R Lang K Hyacinth HI Awosika OO Kissela BM. Early rehabilitation after stroke: a narrative review. Curr Atheroscler Rep. (2017) 19:59. 10.1007/s11883-017-0686-6 29116473 PMC5802378

[16] Wu CM McLaughlin K Lorenzetti DL Hill MD Manns BJ Ghali WA. Early risk of stroke after transient ischemic attack: a systematic review and meta-analysis. JAMA Internal Med. (2007) 167:2417–22. 10.1001/archinte.167.22.2417 18071162

[17] McGirr A Van den Eynde F Tovar-Perdomo S Fleck MP Berlim MT. Effectiveness and acceptability of accelerated repetitive transcranial magnetic stimulation (rTMS) for treatment-resistant major depressive disorder: an open label trial. J Affect Disord. (2015) 173:216–20. 10.1016/j.jad.2014.10.068 25462419

[18] Baeken C Vanderhasselt MA Remue J Herremans S Vanderbruggen N Zeeuws D. Intensive HF-rTMS treatment in refractory medication-resistant unipolar depressed patients. J Affect Disord. (2013) 151:625–31. 10.1016/j.jad.2013.07.008 23896317

[19] Gu SY Chang MC. The effects of 10-Hz repetitive transcranial magnetic stimulation on depression in chronic stroke patients. Brain Stimul. (2017) 10:270–4. 10.1016/j.brs.2016.10.010 27839722

[20] Kim BR Kim DY Chun MH Yi JH Kwon JS. Effect of repetitive transcranial magnetic stimulation on cognition and mood in stroke patients: a double-blind, sham-controlled trial. Am J Phys Med Rehabil. (2018) 89:362–8. 10.1097/PHM.0b013e3181d8a5b1 20407301

[21] Kim KU Kim SH An TG. The effects of repetitive transcranial magnetic stimulation (rTMS) on depression, visual perception, and activities of daily living in stroke patients. J Physical Ther Sci. (2017) 29:1036–9. 10.1589/jpts.29.1036 28626318 PMC5468193

[22] Slotema CW Blom JD Hoek HW Sommer IE. Should we expand the toolbox of psychiatric treatment methods to include Repetitive Transcranial Magnetic Stimulation (rTMS)? A meta-analysis of the efficacy of rTMS in psychiatric disorders. J Clin Psychiatry. (2010) 71:873–84. 10.4088/JCP.08m04872gre 20361902

[23] Duan X Yao G Liu Z Cui R Yang W. Mechanisms of transcranial magnetic stimulation treating on post-stroke depression. Front Neurol. (2018) 12:215. 10.3389/fnhum.2018.00215 29899693 PMC5988869

[24] Currier MB Murray GB Welch CC. Electroconvulsive therapy for post-stroke depressed geriatric patients. J Neuropsychiatry Clin Neurosci. (1992) 4:140–4. 10.1176/jnp.4.2.140 1627974

[25] Wassermann EM Zimmermann T. Transcranial magnetic brain stimulation: therapeutic promises and scientific gaps. Pharmacol Ther. (2012) 133:98–107. 10.1016/j.pharmthera.2011.09.003 21924290 PMC3241868

[26] Paus T Castro-Alamancos MA Petrides M. Cortico-cortical connectivity of the human mid-dorsolateral frontal cortex and its modulation by repetitive transcranial magnetic stimulation. Eur J Neurosci. (2001) 14:1405–11. 10.1046/j.0953-816x.2001.01757.x 11703468

[27] Dubin MJ Mao X Banerjee S Goodman Z Lapidus KA Kang G. Elevated prefrontal cortex GABA in patients with major depressive disorder after TMS treatment measured with proton magnetic resonance spectroscopy. J Psychiatry Neurosci. (2016) 41:E37–45. 10.1503/jpn.150223 26900793 PMC4853214

[28] Nordmann G Azorina V Langguth B Schecklmann M. A systematic review of non-motor rTMS induced motor cortex plasticity. Front Hum Neurosci. (2015) 9:416. 10.3389/fnhum.2015.00416 26257632 PMC4508515

[29] Autry AE Monteggia LM. Brain-derived neurotrophic factor and neuropsychiatric disorders. Pharmacol Rev. (2012) 64:238–58. 10.1124/pr.111.005108 22407616 PMC3310485

[30] Chang WH Shin MA Lee A Kim H Kim YH. Relationship between serum BDNF levels and depressive mood in subacute stroke patients: a preliminary study. Int J Mol Sci. (2018) 19:3131. 10.3390/ijms19103131 30322026 PMC6213140

[31] Li Y Peng C Guo X You JJ Yadav HP. Expression of brain-derived neurotrophic factor and tyrosine kinase b in cerebellum of poststroke depression rat model. Chin Med J. (2015) 128:2926–31. 10.4103/0366-6999.168058 26521792 PMC4756899

[32] Na KS Won E Kang J Chang HS Yoon HK Tae WS. Brain-derived neurotrophic factor promoter methylation and cortical thickness in recurrent major depressive disorder. Sci Rep. (2016) 6:21089. 10.1038/srep21089 26876488 PMC4753411

[33] Phillips C. Brain-derived neurotrophic factor, depression, and physical activity: making the neuroplastic connection. Neural Plast. (2017) 2017:7260130. 10.1155/2017/7260130 28928987 PMC5591905

[34] Yang XR Kirton A Wilkes TC Pradhan S Liu I Jaworska N. Glutamate alterations associated with transcranial magnetic stimulation in youth depression: a case series. J ECT. (2014) 30:242–7. 10.1097/YCT.0000000000000094 24820947

[35] Strafella AP Paus T Fraraccio M Dagher A. Striatal dopamine release induced by repetitive transcranial magnetic stimulation of the human motor cortex. Brain. (2003) 126:2609–15. 10.1093/brain/awg268 12937078

[36] Cho SS Strafella AP. rTMS of the left dorsolateral prefrontal cortex modulates dopamine release in the ipsilateral anterior cingulate cortex and orbitofrontal cortex. PLoS ONE. (2009) 4:e6725–25. 10.1371/journal.pone.0006725 19696930 PMC2725302

[37] Etiévant A Manta S Latapy C Magno LA Fecteau S Beaulieu JM. Repetitive transcranial magnetic stimulation induces long-lasting changes in protein expression and histone acetylation. Scient Rep. (2015) 5:16873. 10.1038/srep16873 26585834 PMC4653621

[38] Chervyakov AV Chernyavsky AY Sinitsyn DO Piradov MA. Possible mechanisms underlying the therapeutic effects of transcranial magnetic stimulation. Front Hum Neurosci. (2015) 9:303. 10.3389/fnhum.2015.00303 26136672 PMC4468834

[39] Hosomi K Morris S Sakamoto T Taguchi J Maruo T Kageyama Y. Daily repetitive transcranial magnetic stimulation for poststroke upper limb paresis in the subacute period. J Stroke Cerebrovasc Dis. (2016) 25:1655–64. 10.1016/j.jstrokecerebrovasdis.2016.02.024 27067882

[40] Guo Z Jin Y Peng H Xing G Liao X Wang Y. Ipsilesional high frequency repetitive transcranial magnetic stimulation add-on therapy improved diffusion parameters of stroke patients with motor dysfunction: a preliminary DTI study. Neural Plasticity. (2016) 2016:11. 10.1155/2016/6238575 27840742 PMC5093297

[41] Guan YZ Li J Zhang XW Wu S Du H Cui LY. Effectiveness of repetitive transcranial magnetic stimulation (rTMS) after acute stroke: a one year longitudinal randomized trial. CNS Neurosci Therap. (2017) 23:940–6. 10.1111/cns.12762 28971620 PMC6492666

[42] Conforto AB Anjos SM Saposnik G Mello EA Nagaya EM Santos W. Transcranial magnetic stimulation in mild to severe hemiparesis early after stroke: a proof of principle and novel approach to improve motor function. J Neurol. (2012) 259:1399–405. 10.1007/s00415-011-6364-7 22173953 PMC4883097

[43] Rossini D Magri L Lucca A Giordani S Smeraldi E Zanardi R. Does rTMS hasten the response to escitalopram, sertraline, or venlafaxine in patients with major depressive disorder? A double-blind, randomized, sham-controlled trial. J Clin Psychiatry. (2005) 66:1569–75. 10.4088/JCP.v66n1212 16401159

[44] Taylor R Galvez V Loo C. Transcranial magnetic stimulation (TMS) safety considerations and recommendations. Transcran Magnet Stimul. (2014) 89:15–30. 10.1007/978-1-4939-0879-0_229338288

